# An F_2_ Barley Population as a Tool for Teaching Mendelian Genetics

**DOI:** 10.3390/plants10040694

**Published:** 2021-04-03

**Authors:** Estela Giménez, Elena Benavente, Laura Pascual, Andrés García-Sampedro, Matilde López-Fernández, José Francisco Vázquez, Patricia Giraldo

**Affiliations:** 1 Department of Biotechnology-Plant Biology, School of Agricultural, Food and Biosystems Engineering, Universidad Politécnica de Madrid, 28040 Madrid, Spain; e.benavente@upm.es (E.B.); laura.pascual@upm.es (L.P.); matilde.lopez@upm.es (M.L.-F.); josefrancisco.vazquez@upm.es (J.F.V.); 2 Institute for Liver & Digestive Health, University College London, Hampstead, London NW3 2QG, UK; andres.sampedro.17@ucl.ac.uk

**Keywords:** genetics education, Mendelian inheritance, qualitative traits, quantitative traits, genetic linkage, molecular markers

## Abstract

In the context of a general genetics course, mathematical descriptions of Mendelian inheritance and population genetics are sometimes discouraging and students often have serious misconceptions. Innovative strategies in expositive classes can clearly encourage student’s motivation and participation, but laboratories and practical classes are generally the students’ favourite academic activities. The design of lab practices focused on learning abstract concepts such as genetic interaction, genetic linkage, genetic recombination, gene mapping, or molecular markers is a complex task that requires suitable segregant materials. The optimal population for pedagogical purposes is an F_2_ population, which is extremely useful not only in explaining different key concepts of genetics (as dominance, epistasis, and linkage) but also in introducing additional curricular tools, particularly concerning statistical analysis. Among various model organisms available, barley possesses several unique features for demonstrating genetic principles. Therefore, we generated a barley F_2_ population from the parental lines of the Oregon Wolfe Barley collection. The objective of this work is to present this F_2_ population as a model to teach Mendelian genetics in a medium–high-level genetics course. We provide an exhaustive phenotypic and genotypic description of this plant material that, together with a description of the specific methodologies and practical exercises, can be helpful for transferring our fruitful experience to anyone interested in implementing this educational resource in his/her teaching.

## 1. Introduction

Given the undoubted importance of genetics in relevant aspects of human lives and activities (medicine or agriculture, among others), an enhanced understanding of its fundamental pillars is necessary to prepare the next generation of scientists and to ensure that life-science students acquire solid knowledge of basic genetic concepts. That is needed for a mindful interpretation of continuous advances in this field and for the appropriate use of genetic applications [1]. Education based on memorization of facts and methods is insufficient in a society and economy based on knowledge. Moreover, several evidences indicate that the information itself is insufficient as an educational objective, and current society requires the use of alternative learning pathways to understand complex concepts and to able to work through and generate new theories, ideas, and products [2].

There are several difficulties in genetics education. Besides the abstract nature of the subject and the specific terminology, the mathematical descriptions of Mendelian inheritance and population genetics are sometimes discouraging, which may lead to the acquisition of misconceptions [3,4]. Improvement strategies in expositive classes can encourage student participation and motivation, but presentation of concepts only through lectures gives many students a superficial understanding of the subject [5]. Following Dopico and co-workers, research in real contexts and environments is a highly motivating and educationally responsible resource for students in modern education [6]. Laboratories, where hands-on experiments can be performed, are not only one of the preferred academic activities for students but also a fruitful learning environment that can be used beyond text-books and lectures as a teaching element of methodological change and educational innovation [7,8,9].

Genetic practices are usually employed with the aim to teach experimental methods such as polymerase chain reaction (PCR and RT-PCR), nucleic acids and protein analysis, etc. However, the inclusion of complex concepts that are common in basic and applied research (i.e., genetic interaction, quantitative inheritance, genetic linkage, statistic for inheritance studies, molecular markers, etc.) is a hard task, and a suitable segregant population is required to address and strengthen those concepts. Several types of plant populations can be used: F_2_, backcross (BC), recombinant inbred lines (RILs), doubled haploid (DH), etc. In a general course on genetics, the optimal population to carry out a genetic study is an F_2_ progeny. In addition to nicely exemplifying Mendel’s original experimental approach, this type of segregant population is extremely useful in explaining different key concepts of genetics (as dominance and epistasis) to students and in teaching additional aspects, particularly concerning statistical analysis (i.e., [9]). However, such a goal makes it necessary to develop an F_2_ consisting of a large number of individuals (150–200 plants), which might require a huge space, either in a greenhouse or in the field. It would also imply having the plants ready to be examined by the students at the right time as the theory classes are developed. This can be especially hard to fit into the academic calendar in addition to requiring a high endowment of materials and human resources. To be able to solve all these problems, a cereal species is the most viable option, as the dry ears can be stored and maintained for successive student generations, allowing for phenotypic studies without the need to cultivate the lines yearly. In this sense, some interesting resources have been developed in maize for teaching purposes [10].

Among cereals, barley possesses several unique features for demonstrating genetic principles: (i) it is a diploid species (2n = 14), with a small genome that is easy to handle [11]; (ii) the barley genome sequence was made available a long time ago [12], and numerous genetic maps and genomic resources are accessible [13]; (iii) it possesses a wide range of phenotypic variation for various traits, particularly grain and spike traits, that are easily scored on dry material; and (iv) it is easy to cross and grow in a green house or field. There is a well-known barley collection, the Oregon Wolfe Barleys (OWB) (https://barleyworld.org/owb; accessed on 17 February 2021), developed several years ago as a teaching resource for understanding the importance and uses of genetic diversity in plants. It was launched at Oregon State University by Dr. Bob Wolfe, who developed the parental lines by systematically crossing recessive alleles into one parent and dominant alleles into the other parent [14,15]. From these parental lines, two different OWB doubled-haploid populations were developed [16,17]. These barley lines provide a highly segregant resource for the construction of genetic maps [18,19,20] and a unique genetic background for mapping of complex traits [21,22]. The OWB populations have been extensively used for teaching aims, and several lesson plans are available at https://barleyworld.org/main/education (accessed on 17 February 2021). We enlarged the pedagogical toolbox by generating an F_2_ population from the cross of the parental lines. The objective of this paper is to present this population as an impeccable model to teach Mendelian genetics. We aim to transfer our experiences with the exhaustive description of the material as well as the specific methodologies and practical exercises carried out in genetics courses at the Universidad Politécnica de Madrid (UPM) to anyone interested in implementing this resource in his/her teaching.

## 2. Results and Discussion

### 2.1. Segregation of Morphological Traits in the Barley F_2_ Population

#### 2.1.1. Qualitative Traits

**Figure 1:**
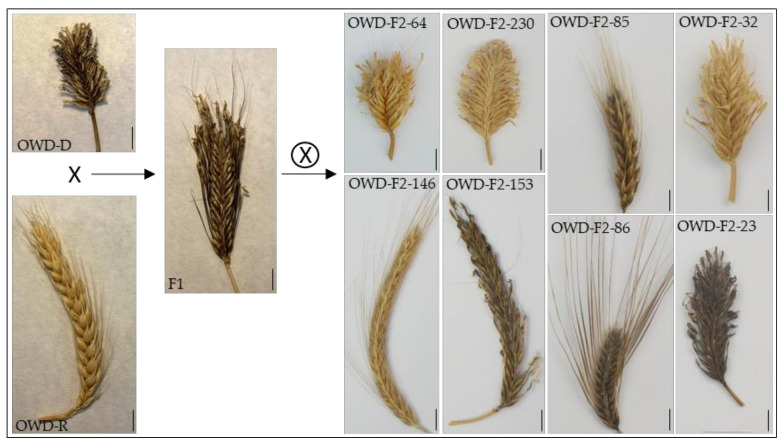
Parental lines (Oregon Wolfe Barleys (OWB)-D and OWB-R), F_1_, and some F_2_ individuals showing the wide range of variability in this population. The scale bars represent 1 cm.

The segregation analysis of morphological qualitative traits selected for this study (see the Materials and Methods section) is presented in Table 1. Among them, type of spike, number of rows, type and length of awns, and type of grain were scored in dry spikes that had been stored when the F_2_ plants reached maturity (Figure 1), while leaf variegation and stem pubescence were directly scored when F_2_ plants were grown in the green house. Some individuals were missed for the stem pubescence phenotype, but a sample size of 256 is still large enough for statistically soundness.

The traits controlled by *Vrs1*, *Nud*, *Zeo*, *Wst,* and *Hsh* behave as expected for a Mendelian monogenic dominant inheritance, segregating in a 3:1 ratio in the population. However, the traits controlled by *Lks2* and *Kap* (length and type of awn) do not follow the expected 3:1 segregation, with the fitting of the type of awn (hooded versus normal) to a 9:7 segregation suggesting more complex genetic control. These two traits, related to the awn morphology, provide an excellent example to introduce the students to non-Mendelian segregations. It is known that the hooded barley phenotype is caused by a mutation in the *Hvknox3* gene (in chromosome 4H), involved in floral evocation [24], but there are epistatic effects involving other genes such as *Lks2* (7H) that code for a transcription factor of the Short Internodes (SHI) family that regulates awn elongation and pistil morphology [25]. In the case of *Lks2*, the classification of awns as long or short can only be made in normal awned spikes (*kapkap*). Hence, an analysis of the character “type of awn” must be re-done with both characteristics (type and length), which classifies the spikes in three phenotypic classes: hooded, normal short, and normal long. This analysis allows us to confirm the segregation 9:3:4 corresponding to a recessive epistasis (Table 2).

#### 2.1.2. Quantitative Traits

**Figure 2:**
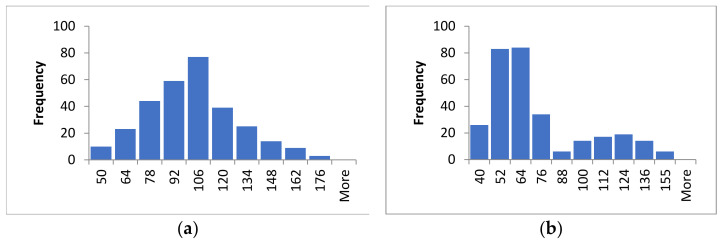
Distribution observed for (**a**) plant height (cm) and (**b**) spike length (mm) in the F_2_ barley population.

Plant height and spike length of the F_2_ plants were measured by the teacher’s team in the green house in 2016 and are provided to students every year since then. The analysis of both traits is presented in Table 3 and Figure 2.

Plant height showed a right-skewed distribution (Figure 2a), while spike length showed a bimodal curve (Figure 2b). The latter can be taken as a representative example of quantitative inheritance when a major locus is involved in trait variation. The coincidence of the barley *Zeo* gene controlling spike compactness, with quantitative trait loci (QTLs) for plant height and spike length, has been reported [16]. In agreement with that, a basic Student’s *t* test demonstrates that mean values are significantly different between F_2_ individuals expressing the dominant and recessive *Zeo* alleles, with the latter being associated with taller plants and longer spikes (for plant height: 87.11 versus 123.80 cm, *t* = 11.28, d.f. = 104, *p* < 0.0001; for spike length: 54.16 versus 111.95 mm, *t* = 25.86, d.f. = 98, *p* < 0.0001). Mean differences are, however, not obtained when the dominant and recessive phenotypes for the other qualitative traits are contrasted (results not shown).

### 2.2. Barley F_2_ Genotypic Description

The segregation analysis of the molecular markers selected for this study (see the Materials and Methods section) is presented in Table 4. Among the simple sequence repeat (SSR) markers, Bmag 0211 and HVM40 behaved as expected for a Mendelian codominant inheritance while Bmac 0310 showed a slight deviation from the expected for a codominant marker. The Knox-dup marker showed a 3:1 segregation, in agreement with the expected for a dominant marker.

As the Knox-dup marker is completely linked to the *Kap* gene, dissection of the genetic interaction can be attempted by the classification of F_2_ individuals not only as hooded, short, or long, but also as dominant or recessive for this gene. The combined analysis is presented in Table 5.

F_2_ individuals carrying the epistatic recessive allele in homozygosis, *lks2lks2*, always develop a short-type awn with independence of the *Kap* genotype. Individuals with homozygous recessive *kapkap* carrying a dominant allele *Lks2* develop a long-type awn. The individuals carrying one dominant allele in each locus develop mostly a hooded awn, but a small proportion (10 out of 179) develop a normal long awn. This suggests that some additional loci can be modulating this complex phenotype (Table 5).

### 2.3. Linkage Analysis

With all the data for qualitative traits and molecular markers, we performed linkage analyses. The *Kap* morphological marker could not be included due to epistasis, but its molecular counterpart, the dominant Knox-dup marker, was used instead. *Lks2* was analysed according to the genetic model previously stablished (Table 5); that is to say, individuals with hooded and normal long awns, assumed to bear the dominant *Lks* allele, composed the dominant phenotype class while individuals with normal short awns represented the recessive *lks* phenotype. The chi-square values for independence are presented in Table 6. All tests including a codominant marker are based upon the 2 × 3 linkage contingency tables instead of the typical 2 × 2 contingency tables used for pairs of dominant traits.

**Figure 3:**
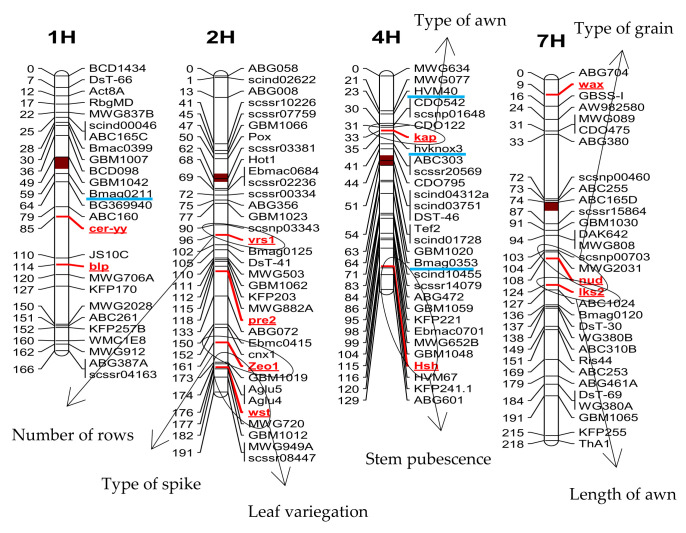
Location of the molecular markers (underlined in blue) and genes controlling the phenotypic traits used in this work on the genetic map of the Oregon Wolfe Barley population available at https://barleyworld.org/owb (accessed on 17 February 2021). In chromosome 4H, HvKnox3 stands for the Knox-dup molecular marker and Bmag0353 points the map location of Bmac 0310.

The analyses revealed three cases of genetic linkage between morphological markers. The best way to calculate the genetic distance (r) between two linked markers in the coupling phase, as is the case in an F_2_ population, is by using the following equation:

r = 1 − √x

where x can be estimated from the second-grade equation:

Nx^2^ + (−a_1_ + 2a_2_ + 2a_3_ + a_4_)X − 2a_4_

in which the a_1_, a_2_, a_3_, and a_4_ values stands for the number of individuals that correspond to the four phenotypic categories of the 2 × 2 F_2_ contingency table, i.e., dominant for both markers (a_1_), dominant for one marker and recessive for the other marker (a_2_ and a_3_), and recessive for both markers (a_4_). These estimations gave a value of r = 0.398 between *Vrs1* and *Zeo* loci and of r = 0.251 between *Zeo* and *Wst* loci. In this population, no linkage was detected between *Wst* and *Vrs1*, which points to *Zeo* as the central locus (Figure 3). A close linkage between *Lks2* and *Nud* (r = 0.10) was also detected.

Regarding the molecular markers, r can be estimated between the marker pairs HVM40 and Knox-dup (r = 0.329) and between Knox-dup and Bmac 0310 (r = 0.375). To calculate these r values, codominant molecular markers can be converted into a dominant marker; thus, the students can use the same equation for all the traits. No linkage could be detected between HVM40 and Bmac 0310, which, following the same reasoning as before, indicates that Knox-dup is the central locus (Figure 3). Our data support genetic linkage between Knox-dup and the *Hsh* locus, with an r value of 0.403. Although these two loci are actually in chromosome 4H, their linkage was unexpected because Bmac 0310, which is located between them, segregates as not linked with *Hsh*. This result could be related to the segregation distortion detected for Bmac 0310 (Table 6). However, there is also an unexpected linkage relationship between Knox-dup (4H) and *Lks2* (7H), which might be derived from the existence of some additional loci modulating awn morphology, as already discussed. It should be noted that all unexpected linkage results involve the Knox-dup marker.

### 2.4. Teaching Experience

This section describes one type of practical exercise that can be developed from the material and data described in this manuscript. It is designed for students of a “genetics” general course at a BSc level but can be adapted for other courses and levels. The exercise must be scheduled once the topics of transmission genetics and molecular markers needed for completing the assignment were covered in the course.

The exercise was organized in groups of around 20 students that attend 4 sessions of 2–3 h each. The practice guideline followed by students can be found as Supplementary Materials.

Session 1: Phenotyping (2 h). This session can be easily set up in a regular classroom. First, the professor explains in detail the characteristics to be scored in the plant material (dry spikes and grains) by the students. The collection of spikes is split in subsets so that the data for the whole F_2_ population are obtained by combining all the subsets’ data. The characteristics recorded by the students are number of rows (2 vs. 6), type of grain (covered vs. naked), type of spike (dense vs. lax), and type of awn (hooded vs. normal). The students, in pairs, must characterise the phenotypes of the F_2_ spikes assigned (in our case, 40–50 F_2_ individuals) and must record the observations in an Excel datasheet. At the end of session 1, the professor obtains a file with the phenotype records of the complete collection.

Session 2: Genetic analysis (3 h). In this session, which must be held in a computer room, the professor guides the students in genetic analysis. This training is essential for successful completion of the final report. Several points are covered and discussed:

1. Data acquisition: the phenotype profiles recorded by the students are compared with that recorded by the teaching team (“official phenotyping”).
2. Segregation analysis of single traits: for this and further analyses, the professor provides the data for the two additional qualitative traits measured in grown plants (stem pubescence and leaf variegation). The students check if the traits behave as expected assuming a model of genetic control by one locus with two alleles and complete dominance. They must employ Excel for data management and χ2 analysis. There is only one trait, type of awn, that does not behave as expected (Table 1). All of the groups discuss what can be happening with this trait. The professor leads the students to understand and to conclude that its genetic control may be an epistasis and provides the data for the length of the spike. At this moment, it can be useful to give again the spikes to the student so they can see that the “normal awn” phenotype can be subclassified as long awn and short awn. This trait is not easy to score in F_2_ plants; therefore, in our experience, it is more convenient to give the data to the students. With the combined data, the students must check if the segregation observed really corresponds with an epistasis.
3. Linkage analysis: students, in pairs, perform the linkage analysis for all combinations of two of the seven traits, estimate the recombination fraction, and create a genetic map. The professor leads the students to understand that, despite no linkage detected between *Wst* and *Vrs1* genes, both show linkages with *Zeo1* (Table 6), which indicates that these three genes are placed in the same linkage group. In this case, students can calculate not only the recombination fraction but also interference and coincidence coefficients. Likewise, it is important to discuss why they cannot estimate r with the *Kap* gene, even if they detect genetic linkages with other genes. Students’ conclusions can be used also to contrast with those based on official phenotyping, which is especially useful if phenotyping errors may have resulted in misleading outcomes.

Session 3: Molecular markers I (2 h). This session must be performed in a laboratory. The students amplify two molecular markers, Knox-dup and Bmac 0310, using PCR. First, the professor explains the fundamentals of PCR and how the reaction works. Then, each pair of students is provided with DNA from 6 F_2_ individuals and from the parental lines, and all the reagents and materials needed for the experiment. In order to promote autonomous work, the students must design the experiment, including the calculation of the reagents’ volumes in the PCR mix, and must perform it on their own.

Session 4: Molecular markers II (3 h). The students analyse the results of the PCR by agarose gel electrophoresis. The inclusion of a dominant and a codominant marker allow them to discuss the differences in the results. Genotyping data must be included in the Excel datasheet. At the end of session 4, the professor obtains a file with the genotype data for all the collections and make it available to all the students. For a more complete analysis, the genotypic profile of the F_2_ individuals for two additional SSR markers (HVM40 and Bmag 0211) could be included in the datasheet.

Results report (3 h personal work). Once the sessions are completed, the students, in pairs, must fill out a report. In this document, they must present: 1. the study of individual segregation of the four molecular markers, 2. the linkage analysis in pairs for the four molecular markers, 3. the linkage analysis in pairs between the morphological traits and the molecular markers, and 4. a conclusion of the analyses. It is worth noting that the analyses requested in item 3 are not based on students’ recorded data but on the official phenotypic and genotypic data provided by the professor.

Extra session: Class discussion (1 h). Once all the reports have been submitted and reviewed, the professor may schedule an extra session in which the more common troubles faced by the students can be discussed.

Additional exercises can be carried out with the F_2_ population in order to study the quantitative traits.

### 2.5. Learning Experience

During the past 5 years, about 500 students have completed this practical activity. Student accuracy in phenotyping is low, with 70–80% of the raw forms needing correction. On the contrary, molecular marker practices and genotyping are usually easier than phenotyping for students. Most of the students carry out the PCRs adequately, without contamination or false-negative results.

Personalized discussion with each student during the first practical session helps to reduce the error rate. The number of rows is the easiest trait to be assessed by students, with the lowest rate of mistakes. The type of grain and the type of awn usually show more errors; however, the mistakes are generally small and do not affect the results obtained in segregation and linkage analyses. The type of spike (dense or lax) is the most difficult trait to be scored for students in F_2_ individuals because, in some plants with dense spikes, the phenotype is not as extreme as in the homozygous OWB-D parent (see OWB-F2-85 in Figure 1). Thus, the number of mistakes can be large enough to significantly modify the results of the genetic analyses. This point allows to discuss with the students the importance of finely performing the phenotypic studies.

With the aim to know the profile of students who perform the practices, their opinion about practice exercises in general, and genetics practices in particular, 73 students were surveyed during the 2019–2020 course. The age range of the students surveyed varied between 18 and 55 years, although the majority were 19 or 20 years old since they were in their second course of the degree (Figure S1). These students belonged to the biotechnology degree (55), or agrarian sciences and bioeconomy degree (18) (Figure S1); in both, the genetic course was placed in the second course. Most of students were females (46 vs. 27) and claimed that they have prior knowledge of genetics and that they liked genetics and practical exercises (Figure S1).

According to the survey (see supplementary Table S1), less than half of the students really understood the importance of using a cereal to perform this practice exercise. In agreement with that, more than half of the students thought that this practices could be carried out with some type of horticultural plant, without understanding that fresh fruits from horticultural cannot be conserved and would not allow us to schedule and to perform the practice satisfactorily in the same conditions.

In addition, near 90% of students claimed to understand the benefits of using a F_2_ population in the practices. However, near 22% of them thought that an F_1_ population or test cross would be just as suitable as F_2_ and 27.4% of students thought that an F_1_ population and test cross could be used in these practices. This result evidences that many students do not understand that the complete study described in this project can only be carried out with an F2 population (Table S1).

When the students were asked about practice exercises in general, the majority of them thought that bachelor’s practices allow them to become familiar with the experimental techniques (almost 90% mostly or completely agree) and facilitates their understanding of the related subject (around 80% mostly or completely agree). A similar percentage of students claimed that genetics practices make understanding concepts of genetics easier, although only 45% believed that genetics practices are useful to pass the subject (Table S1). This last point was also evidenced when the students were asked about which lecture topics were implicated in this practice exercise, and only 22 out of 55 biotechnology students and 4 out of 18 students of agrarian sciences and bioeconomy answered correctly. The lack of connection between practices and theory perceived by some students is very common. Therefore, professors must continue to put in huge efforts to connect both kinds of teaching so that students understand that the practices exercises are based on real projects designed according to theoretical concepts of genetics that are studied in theoretical classes.

## 3. Materials and Methods

### 3.1. Barley F_2_ Generation

#### 3.1.1. The Plant Material

A *Hordeum vulgare* L. F_2_ population was generated from the cross of the recessive (OWB-R) and dominant (OWB-D) spring barley stocks (Figure 1) previously described [14,15]. One spike from two OWB-R plants were hand emasculated and pollinated with the pollen of OWB-D plants. Crosses were made in May 2014, and the seven F_1_ seeds obtained were sown in November 2014 in a greenhouse (1 seed/pot) at the School of Agricultural, Food, and Biosystems Engineering of UPM. After self-pollination of the F_1_ plants, more than 500 F_2_ seeds were obtained. From autumn 2015 to summer 2016, 303 F_2_ plants were grown in the greenhouse with a manually controlled window system to avoid extreme indoor temperatures. Flag leaf samples were taken for DNA extraction, which was carried out by professors using the cetyl trimethylammonium bromide (CTAB) method, and 6–8 mature spikes of each plant were collected and stored in labelled plastic bags, which also contained a few harvested grains of the corresponding F_2_ plant. The remaining F_2_ seeds are kept in a dry environment at 4 °C and will replace the actual set when needed.

#### 3.1.2. Phenotyping of Morphological Traits

As mentioned before, the OWB-D and OWB-R lines differ from each other for many morphological characteristics whose segregation can be easily monitored in a segregant population. In this type of exercise, it is very important to use a coherent set of traits easy to handle by the students. Therefore, from all the possible traits, we selected the nine that are described in Table 7. Five traits (type of spike, number of rows, type and length of awns, and type of grain) can be directly scored in dry spikes by students, while the remaining four (leaf variegation, stem pubescence, plant height, and spike length) must be scored in growing plants.

The phenotypes for the traits that had to be assessed in growing plants were recorded in the greenhouse during the academic year 2015–16 by the team involved in the development of this teaching resource. In the following courses, these data are given to the students to complete the data set for further analyses.

#### 3.1.3. Genotyping of Barley F_2_ Population

**Figure 4:**
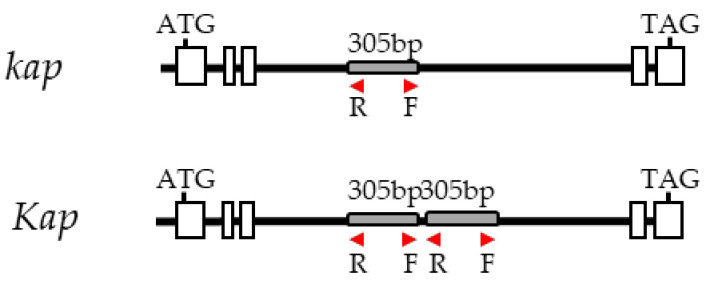
Schematic representation of the dominant marker Knox-dup. R: reverse primer, F: forward primer, black lines: introns, white boxes: exons, grey boxes: the 305 bp polymorphic fragment present in intron 4.

There is a lot of information about simple sequence repeats (SSR) and single-nucleotide polymorphisms (SNPs) markers in the OWB population (https://barleyworld.org/owb/data; accessed on 17 February 2021). For didactic purposes, three SSR markers selected from the literature and one PCR-based dominant marker were chosen. This latter, the Knox-dup marker, was developed in house for allelic discrimination of the *Hvknox3* gene that is located on the short arm of barley chromosome 4 [24]. The dominant allele (*Kap*) of this gene is responsible for the hooded phenotype and differs from the recessive allele (*kap*) in a tandem duplication of 305 bp located in intron IV of this allele. The oligonucleotides design is shown in Figure 4.

**Figure 5:**
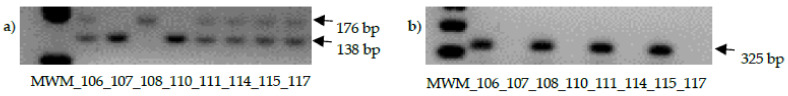
Electrophoresis gel for analysing PCR products from codominant marker Bmac0310 (**a**) and dominant marker Knox-dup (**b**) in eight individuals of the F_2_ population. MWM: molecular weight marker.

The inclusion of a dominant marker is useful in explaining the differences in the analysis of both types of molecular markers (dominant and codominant) and helps the student understand the advantages and disadvantages of their use (Figure 5). Three out of the four markers employed were mapped in chromosome 4H (Table 8), which is relevant for linkage analysis and genetic dissection of the epitasis.

## 4. Conclusions

In this study, an F_2_ population of barley was generated from the two parental lines of the OWB collection. An F_2_ is the most suitable and complete population to perform the study of complex genetic concepts, such as dominance, epistasis, and linkage, and to carry out segregation, linkage, and genetic interaction analyses. Many educational institutions maintain *Drosophila melanogaster* mutant stocks to develop F_2_ populations for genetics practical teaching [26,27]. In our view, utilizing a cereal species as the working organism has several advantages, including the possibility to isolate DNA for genotyping of the F_2_ individuals during the plant growing cycle. Dry material can be kept during long periods of time, traits easily be phenotyped in dry ears, and grains can be selected in order to design the experiments to be performed by students. Among cereals, barley is the best candidate because it has a diploid genome and numerous genetic resources are available. The generation of the barley F_2_ population presented here has allowed for implementation of the practical exercise described here for several years (see Supplementary Materials for a detailed guideline). Furthermore, a collection of images of the stored spikes and grains of each F_2_ individual has recently served as an online phenotyping resource for a group of students that had to follow sessions 1 and 2 from home because of the COVID-19 pandemic.

With accomplishment of the full exercise, the students achieved the following goals: (i) acquire the methodologies for data collection, treatment, and analysis to study the genetic control of qualitative traits and to analyse the existence of genetic linkage between two loci; (ii) acquire the basic knowledge to analyse molecular markers in the laboratory (amplification and electrophoretic analysis of DNA sequences); and (iii) understand the different types of molecular markers and their uses in genetic linkage analysis.

The development of this practice exercise, placed in a context similar to a real research project, aimed to improve learning of complex genetic concepts by students. However, a survey of the students that followed the practice in 2019–2020 indicated that some of them neither understood the importance of using an F_2_ population nor were able to successfully associate the exercise performed with some concepts that are studied in theoretical classes of genetics. Clearly, professors must continue making educational efforts to connect both types of teaching.

## Figures and Tables

**Table 1:** Segregation of morphological traits in the barley F_2_ population. The morphological markers are designated following the nomenclature for barley genes described by Francowiak [23].

Trait	Gene	Dominant	Recessive	N	χ2 _3:1_	*p*	χ2 _9:7_	*p*
Type of spike	*Zeo*	229	74	303	0.05	n.s.		
Number of rows	*Vrs1*	230	73	303	0.13	n.s.		
Type of awn	*Kap*	169	133	302	58.39	***	0.01	n.s.
Length of awn	*Lks2*	56	77	133	76.75	***		
Type of grain	*Nud*	237	65	302	1.95	n.s.		
Leaf variegation	*Wst*	237	66	303	1.67	n.s.		
Stem pubescence	*Hsh*	186	70	256	0.75	n.s.		

***: *p* < 0.001, n.s. (non-significant): *p* > 0.05, N: total number of individuals. The specific phenotypes (dominant and recessive) for each trait are indicated in the Materials and Methods section.

**Table 2:** Segregation by type of awn in the barley F_2_ population.

*Kap/Lks2*						
Phenotype	Hooded Awn	Normal, Long Awn	Normal, Short Awn	N	χ2 _9:3:4_	
Individuals	169	56	77	302	0.14	n.s.

n.s. (non-significant): *p* > 0.05; N: total number of individuals.

**Table 3:** Summary of the quantitative traits in barley F_2_ population.

Trait	Mean	SE	SD	Range	Minimum	Maximum	N
Plant Height (cm)	96.11	1.53	26.57	135	36	171	303
Spike length (mm)	68.27	1.64	28.54	127	28	155	303

SE: standard error, SD: standard deviation, N: number of individuals.

**Table 4:** Segregation of select molecular markers in the barley F_2_ population.

Marker	Homozygous OWB-D Allele	Heterozygous	Homozygous OWB-R Allele	N	Expected Ratio	χ2	*p*
Bmac 0310	57	167	76	300	1:2:1	6.26	*
Bmag 0211	80	130	88	298	1:2:1	5.28	n.s.
HVM40	63	160	78	301	1:2:1	2.69	n.s.
Knox-dup	230		73	303	3:1	0.13	n.s.

*: *p* < 0.05, n.s. (non-significant): *p* > 0.05; N: total number of individuals.

**Table 5:** Segregation of awn phenotypes combined with molecular marker Knox-dup.

Genotype	*Kap_ Lks2_*	*kapkapLks2_*	*Kap_lks2lks2*	*kapkaplks2lks2*	N	χ2 _9:3:3:1_	
Awn phenotype	Hooded/Normal	Normal					
		Long	Short	Short			
Individuals	169 + 10 ^#^	46	50	27	302	6.76	n.s.

^#^ See the text. n.s.: nonsignificant, N: total number of individuals.

**Table 6:** Linkage analysis of the molecular and morphological markers in the barley F_2_ population.

*Gene/Marker*	*Lks2*	*Nud*	*Zeo*	*Wst*	*Hsh*	Knox-dup	Bmac 0310	Bmag 0211	HVM40
*Vrs1*	n.s.	n.s.	6.53 *^a^	n.s.	n.s.	n.s.	n.s.	n.s.	n.s.
*Lks2*		162.93 ***^a^	n.s.	n.s.	n.s.	6.69 *^a^	n.s.	n.s.	n.s.
*Nud*			n.s.	n.s.	n.s.	n.s.	n.s.	n.s.	n.s.
*Zeo*				50.25 ***^a^	n.s.	n.s.	n.s.	n.s.	n.s.
*Wst*					n.s.	n.s.	n.s.	n.s.	n.s.
*Hsh*						5.42 *^a^	n.s.	n.s.	n.s.
Knox-dup							15.47 ***^b^	n.s.	21.92 ***^b^
Bmac 0310								n.s.	n.s.
Bmag 0211									n.s.

Chi-square test indicating significant differences with the expected values assuming independent inheritance are marked in bold. *: *p* < 0.05, ***: *p* < 0.001, n.s. (non-significant): *p* > 0.05; ^a^: 1 degree of freedom, ^b^: 2 degrees of freedom.

**Table 7:** Phenotypic characteristics of the F_2_ parental homozygous lines, OWB-D and OWB-R, for the qualitative and quantitative barley traits selected to be analysed. The morphological markers are designated following the nomenclature for barley genes described by Francowiak [23]. For plant height and spike length, the ranges of values recorded in 5 seasons are given.

Material	Trait	OWB-D Phenotype	OWB-R Phenotype
Dry spikes	Type of spike	Zeo = dense spike	zeo = lax spike
	Number of rows	Vrs1 = two-rowed spike	vrs1 = six-rowed spike
	Type of awn	Kap = hooded awn	kap = normal awn
	Length of awn	Lks2 = long awn	lks2 = short awn
	Type of grain	Nud = covered caryopsis	nud = naked caryopsis
Growing plants	Leaf variegation	Wst = non variegated leaf	wst = variegated leaf
	Stem pubescence	Hsh = hairy leaf sheath	hsh = non hairy leaf sheath
	Plant height	49–96 cm	67–122 cm
	Spike length	35–45 mm	91–116 mm

**Table 8:** Molecular markers selected for genotyping of the barley F_2_ population.

Marker	Class	Oligo Forward	Oligo Reverse	Tm	D Allele	R Allele	Chr
Bmac 0310	SSR	CTACCTCTGAGATATCATGCC	ATCTAGTGTGTGTTGCTTCCT	55 °C	176 pb	138 pb	4
Bmag 0211	SSR	ATTCATCGATCTTGTATTAGTCC	ACATCATGTCGATCAAAGC	55 °C	187 pb	198 pb	1
HVM40	SSR	CGATTCCCCTTTTCCCAC	ATTCTCCGCCGTCCACTC	55 °C	175 pb	146 pb	4
Knox-dup	PCR	CCATGTTGCTGTATTTTGCG	ACTGCACTGCAACTGGTCAG	60 °C	325 pb	-	4

Chr: chromosome, D allele: allele presents in the OWB-D parental line, R allele: allele presents in the OWB-R parental line.

## Data Availability

The full set of phenotype and genotype data of the F2 population described here will be provided upon request.

## Supplementary Materials

The following are available online at https://www.mdpi.com/article/10.3390/plants10040694/s1, Appendix A: Practice guideline followed by students to perform this teaching experience, Figure S1: Students’ profile, Table S1: Opinion of students about genetics practices.
