# Supplementary material for: An F2 Barley Population as a Tool for Teaching Mendelian Genetics
Source: Plants (Basel). 2021 Apr 3;10(4):694. doi: 10.3390/plants10040694 (PMC8066651; doi:10.3390/plants10040694)
Supplement: Supplementary file 1 [file plants-10-00694-s001.zip › Appendix A_v2.pdf]

Genetic analysis of morphological characters and molecular markers in a barley F_2 population (*Hordeum vulgare* L.)

The **objectives** of this practical exercise are:

1. To become familiar with data collection, data treatment and data analysis used in the study of the genetic control of qualitative traits and to be able to estimate the existence of genetic linkage between two loci.
2. To become familiar with basic methodologies for amplification and separation of DNA fragments.
3. To understand the usefulness of different classes of molecular markers (dominant *vs* codominant).

The exercise will be carried out with an $\mathbf{F_2}$ progeny obtained from the cross between two barley inbred lines, the **OWB-Dom** and the **OWB-Rec**, developed at the University of Oregon (USA). These lines differ for many morphological characters, for which the OWB-Dom line is homozygous for the dominant alleles and the OWB-Rec line is homozygous for the recessive alleles. The characteristics of these parental lines, as well as some of the mapping populations developed from them, can be consulted in: http://barleyworld.org/oregonwolfe. The F_2 population has been developed in the Genetics unit of the ETSIAAB-UPM, following the classic crossing scheme:

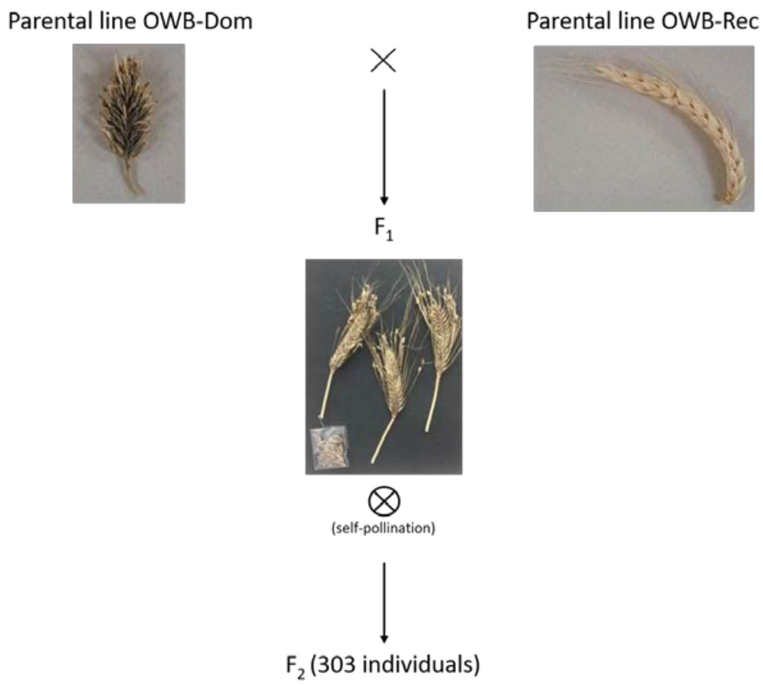

The practical exercise is divided in 4 sessions. In <u>session 1</u>, the study of morphological characters will be carried out on a sample of around 200 individuals of this F_2. In <u>session 2</u>, the quality of the phenotypic observations will be reviewed in detail, and the segregation and linkage analyses of qualitative traits will be done, using appropriate statistical tools. In <u>session 3</u> and <u>session 4</u>, F_2 individuals will be genotyped for molecular markers, and students will perform the linkage analysis between molecular markers and phenotypic traits. Finally, they will sum up their results in a report.

SESSION 1. Evaluation of morphological characters

In this practice, 4 barley characters observable in dry spike or grains will be phenotyped in the F_2 plants.

- Number of rows (*Vrs1* locus): two-rowed spike (dominant), six-rowed spike (recessive)
- Type of spike (*Zeo* locus): dense spike (dominant), lax spike (recessive)
- Type of awn (*Kap* locus): hooded awn (dominant), normal awn (recessive)
- Type of grain (*Nud* locus): covered caryopsis (dominant), naked caryopsis (recessive)

Students will work in groups. The data obtained from the phenotyping study will be included in an excel table provided to students (“**Phenotypes&GenotypesF2.xlsx**”). This table already contains the phenotype of each F_2 individual for another two morphological traits, only observable in living plants, which were evaluated during the growth phase of the F_2 plants in the greenhouse.

- leaf variegation (*Wst* locus): non-variegated leaf (dominant), variegated leaf (recessive)
- leaf pubescence (*Pub* locus): pubescent (dominant), non-pubescent (recessive)

These traits will be also included in the data analysis. Once the table is filled with the data of the 4 traits evaluated in class, each group will send it to the teacher before the indicated deadline.

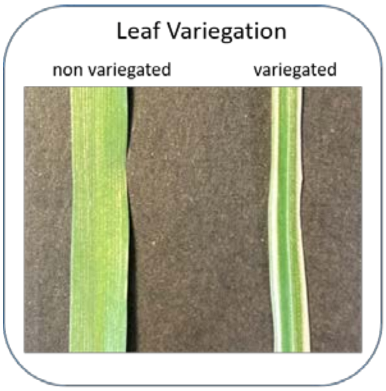

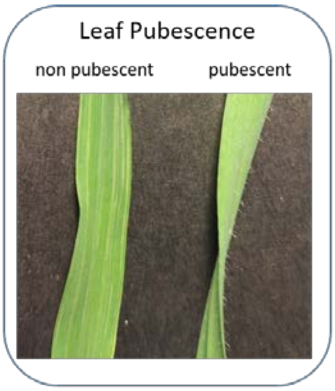

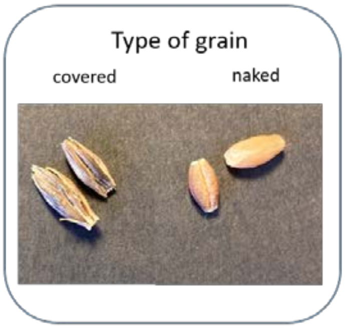

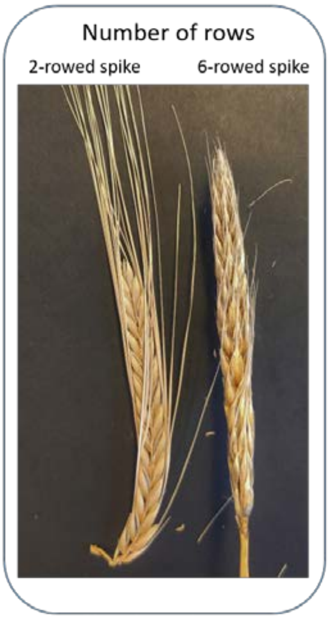

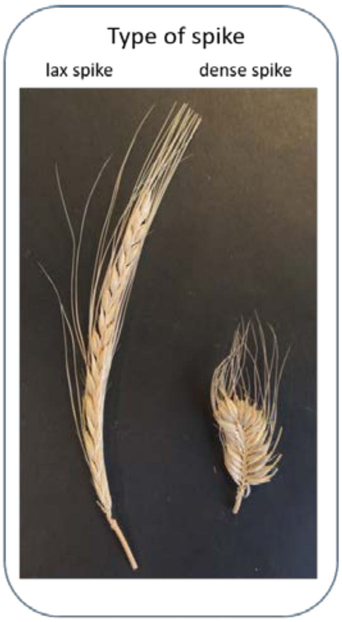

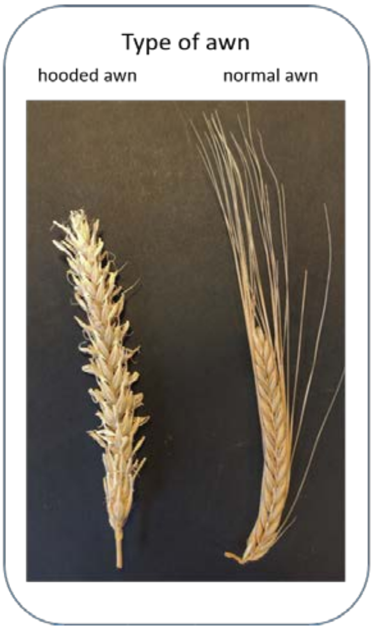

Session 2: Genetic analysis of phenotypic data

During this class session, the data provided by each group will be reviewed in detail and the results obtained will be put in common.

Then, the following analyses will be carried out:

- Segregation analysis of traits: segregation will be analyzed for each of the 6 morphological characters, checking by χ^2 test if the distribution of phenotypes observed in the F_2 [OWB-Dom x OWB-Rec] matches the expected for a character controlled by a locus with two alleles showing complete dominance.

Trait 1	Dominant	Recessive	χ^2	Conclusion
N Observed				
N Expected				

- Genetic linkage analysis: A linkage analysis for all combinations of two of the six traits will be performed in order to determine if there is linkage between their respective loci. Linkage analysis will be done using the χ^2 contingency test. This test compares the observed results with the expected according to the null hypothesis, that assumes the independent segregation of both loci in the F_2 progeny. In those cases where linkage is detected, the genetic distance between the loci involved will be estimated if possible (i. e., if monogenic inheritance was formerly demonstrated for the two corresponding traits).

	**Trait 1**				
**Trait 2**	Dominant	Recessive	N	χ^2	**Conclusion**
Dominant	Oi:<br>Ei:	Oi:<br>Ei:			
Recessive	Oi:<br>Ei:	Oi:<br>Ei:			
N					

Session 3: DNA sequences amplification by PCR

AIM

The aim of this practice is to genotype for selected molecular markers the F_2 barley population that was phenotyped in Practice 1.
To do so, the PCR (Polymerase Chain Reaction) technique will be used for analyzing two different kinds of molecular markers.

SHORT DESCRIPTION OF THE TECHNIQUE

POLYMERASE CHAIN REACTION (PCR)

PCR is a laboratory technique used to **amplify** a DNA fragment of interest, meaning, making multiple copies of it.

Steps

- **Denaturation:** double-stranded DNA templates are heated to separate the strands
- **Annealing:** primers bind to flanking regions of the target DNA
- **Extension:** DNA polymerase extends the 3′ end of each primer along the template strands

Components

- **Template DNA**
- **Taq Polymerase**: is a heat resistant enzyme that synthesizes new strands of DNA complementary to the target sequence, by incorporating nucleotides, to extend primers at their 3′ ends in the 5′ to 3′ direction
- **Primers**: designed to bind sequences that flank the region of interest in the template DNA
- **dNTPs** (tri-phosphate nucleotides): 4 basic nucleotides, dATP, dCTP, dGTP, and dTTP
- **Buffer**: provides a suitable chemical environment for Taq's activity (pH and salts)
- **$MgCl_2$**: cofactor for activity of DNA polymerases, allowing dNTPs' incorporation during polymerization

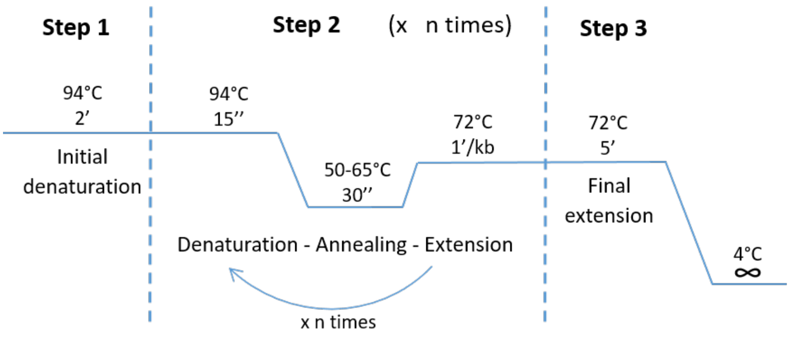

The amplification of the sequences requires rapid changes in temperature. For this, we will use a thermal cycler with a microprocessor that allows to program the timing of temperature changes and the desired number of cycles.

MOLECULAR MARKERS

A) Marker knox-Dup: Dominant marker

This marker is based on a polymorphism present in the *HvKnox* gene, located on chromosome 4 and responsible of the hooded awn phenotype. The dominant allele (D) bears a duplication of 305 bp, not present in the recessive allele (R), that was used for the design of the dominant marker *knox-Dup* according to the following scheme.

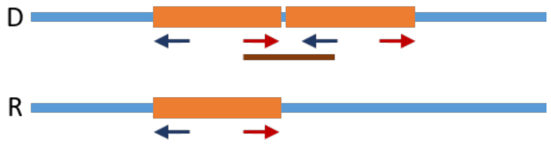

Primers have been designed in a way that PCR amplification can only occur if the dominant allele is present, when a 325pb amplicon is obtained. Marker *knox-Dup* is a dominant marker since it makes not possible to differentiate dominant homozygous individuals from heterozygous individuals since in both cases an amplicon is detected.

B) Marker Bmac310: codominant marker ("Simple Sequence Repeats" or microsatellite)

Microsatellites (SSRs) are DNA sequences in which a di-, tri- or tetra-nucleotide is repeated in tandem numerous times. The variation in the number of repetitions, and not the repeated motif itself, creates the different alleles. An SSR is useful as a molecular marker because the flanking regions are usually highly conserved and can be used for primers design. SSRs are codominant markers, since two different alleles can be distinguished by size in a heterozygous individual.

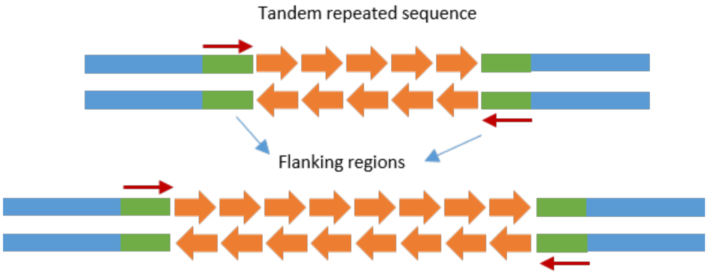

In this practice, the SSR marker *Bmac310* located on chromosome 4 will be amplified. The dominant allele produces an amplicon of 176 bp and the recessive allele produces an amplicon of 138 bp.

PRIMERS SEQUENCES

Marker	Primer name	Sequence 5'-3'	Ta °C
Knox-dup	DupF	CCA TGT TGC TGT ATT TTG CG	60
	DupR	ACT GCA CTG CAA CTG GTC AG	
Bmac310	Bmac310F	CTA CCT CTG AGA TAT CAT GCC	55
	Bmac310R	ATC TAG TGT GTG TTG CTT CCT	

PCR PROTOCOL

NOTE: Work with gloves, and it is very important to preserve all the PCR reagents on crushed ice and to work on it.

1. Set up of the PCR mix: Each reaction must contain the following reagents at the final concentrations indicated in the table. Calculate the reagent volumes for one PCR reaction and for the PCR mix needed to analyze 6 F_2 individuals and the parental lines (mix × 9).

Stock reagents	***Final concentration***	**× 1**	**× 9**
**Reaction Buffer (10×)**	1x		
**$MgCl_2$ (25mM)**	2 mM		
**dNTPs (2.5 mM)**	200 µM		
**Forward Primer (10 µM)**	0.5 µM		
**Reverse Primer (10 µM)**	0.5 µM		
**Taq Polymerase (1 U/µl)**	1 U		
**H_20**	-		
**DNA (50 ng/µl)**	-	2 µl	
**Final Volume**		25 µl	

2. Close the tubes tightly and put them in the thermal cycler.
3. Start the PCR program that corresponds with the primer pairs used.

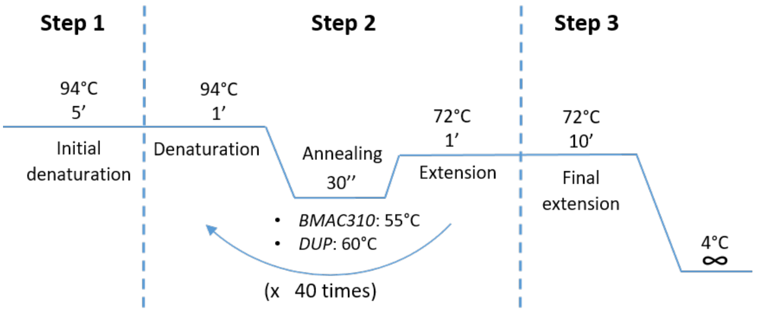

4. Once the program ends, keep the tubes at 4 °C or carry out the corresponding electrophoresis to see the result.

Session 4: DNA electrophoresis in agarose gels

AIM

The aim of this practice is to analyze the result of the PCRs performed in the previous session by means of agarose gel electrophoresis, a widely used technique to separate nucleic acids of different molecular weight.

SHORT DESCRIPTION OF THE TECHNIQUE

The physical principle of agarose gel electrophoresis is the mobility of charged molecules through an agarose matrix under an electric field. DNA fragments, negatively charged, will migrate from anode to cathode, and their mobility will be determined by their molecular weight, regardless of their sequence. This technique is based on the different mobility of molecules due to their molecular weight and the agarose matrix pore size. Thus, smaller fragments will migrate faster than larger ones, which will stay trapped in the pores.

ELECTROPHORESIS and GEL BUFFER: TAE (- Tris - Acetate - EDTA)	AGAROSE GEL (- Agarose powder - TAE Buffer - Gel Red/BrEt/...)	LOADING BUFFER (- 30% Glycerol - 0,25% bromophenol blue - 0,25% xylencianol)
TAE stands for its components (Tris, Acetate and EDTA) and has buffering functions.<br>Electrophoresis tank buffer and gel buffer must be the same.<br>TAE preserves the pH during gel electrophoresis and maintains DNA negatively charged, allowing them to run through the gel, from cathode (-) to anode (+).<br>It also protects DNA from enzymatic activity and from H^+ ions resulting of H_2O hydrolisis.	Agarose gels are used for DNA fragment separation on the basis of their molecular weight.<br>Agarose gel concentration depends on the size of the DNA molecules to be separated. Concentration will depend on fragment size, requiring lower concentration (1%) to separate bigger fragments, and higher concentration (>2%) for the smaller ones.	Loading buffer has 3 main functions when added to samples:<br>- To increase their density, preventing them from coming out of the wells.<br>- To add color, facilitating sample loading in the gel.<br>- To make visible the electrophoresis front position, as it is electrically charged and therefore migrates with the samples through the gel.

PROTOCOL

1. Weigh the agarose needed to make a 50 ml agarose gel at 1% concentration.
2. Pour the agarose into a 100 ml Erlenmeyer flask and add the 1× TAE (prepared from the 10× stock).
3. Heat carefully in the microwave avoiding boiling.
4. Remove the solution from the microwave when it begins to boil. Stir gently and wait for it to cool down to 50 °C approximately. Finally, add 1 µl of GelRed or ethidium bromide (BrEt) (which causes fluorescence in DNA bands when gels are placed under UV light) and stir gently again for complete mixing.

CAUTION! *Both products, GelRed and BrEt are carcinogenic; meaning they intercalate in the DNA. Use of gloves is mandatory.*

5. Slowly pour the agarose into the gel tray (which must be well levelled) and remove any bubbles with a tip. Place the combs carefully, watching that they do not touch the bottom. Let the agarose cool until gelification.

6. Prepare the samples to be analyzed by adding 5 µl of Loading Buffer to each PCR reaction.
7. Once the gel is ready, place it in the buffer tank, and remove the combs.
8. Insert the prepared samples, taking care not to damage the wells, and noting the insertion order. In addition to the samples, markers for both size and quantity of DNA are also loaded on the gel.

Comparing the migration distance and the signal intensity between our samples and the markers, we can estimate the size and concentration of the PCR products.

9. Connect the power supply to the electrodes and turn on, adjust the voltage to 60-100 V. Wait until the samples come out of the wells.
10. Once the samples have migrated enough, stop the electrophoresis and remove the gel from the buffer tank.
11. View on the UV transilluminator and take a picture.

CAUTION! *Protect yourself from UV light.*

In addition to the PCR results obtained by each student in the PCR reactions preformed in practice 3, pictures of additional agarose gels will be provided to the students so that all they can complete the genotyping of the two markers in the entire barley F_2 population.

FINAL REPORT

With the genotypic and phenotypic information of the F_2 population obtained in sessions 1, 3 and 4, students, by pairs, will present a final report that include the following analyses:

- Analysis of individual molecular marker segregation

- Linkage analysis: linkage analysis for the two molecular markers will be performed in order to determine if there is any linkage between their respective loci. The analysis will also include linkage analyses between each of the six morphological markers studied in previous practices and each of the two molecular markers, *Bmac310* or *Knox-Dup*. In those cases where linkage is detected, the genetic distance between the involved loci will be estimated.
