# Supplementary material for: An F2 Barley Population as a Tool for Teaching Mendelian Genetics
Source: Plants (Basel). 2021 Apr 3;10(4):694. doi: 10.3390/plants10040694 (PMC8066651; doi:10.3390/plants10040694)
Supplement: Supplementary file 1 [file plants-10-00694-s001.zip › Table S1.pdf]

Table S1. Opinion of students about genetics practices

	Have you understood the importance of using a cereal in Genetics practices?	Could this practice be carried out with some kind of horticultural?	Do you think that the use of an F2 population facilitates the understanding of the subject taught in theory?	Do you think that another type of population (F1 or test cross) would be just as suitable for carrying out the practices?	Do you consider that Bachelor's practices are designed to become familiar with techniques related to the subject taught?	Do Bachelor´s practices facilitate the understanding and learning of the related subject?	Have you understood the concepts of Genetics better thanks to genetic practices?	Do you consider that the Genetics practices have been useful to pass the subject?
NA	5.48	5.48	0.00	12.33	0.00	1.37	0.00	6.85
LA	13.70	30.14	2.74	32.88	4.11	4.11	4.11	13.70
MA	30.14	43.84	6.85	27.40	6.85	15.07	20.55	27.40
MsA	35.62	5.48	45.21	17.81	64.38	52.05	49.32	24.66
CA	13.70	4.11	43.84	4.11	24.66	27.40	26.03	20.55
N/A	1.37	10.96	1.37	5.48	0.00	0.00	0.00	6.85

Data are given in percentage. Do not agree (NA), Little agree (LA), Moderately agree (MA), Mostly agree (MsA), Completely agree (CA), Not available (N/A).
