# Supplementary figures and images for: An F2 Barley Population as a Tool for Teaching Mendelian Genetics
Source: Plants (Basel). 2021 Apr 3;10(4):694. doi: 10.3390/plants10040694 (PMC8066651; doi:10.3390/plants10040694)

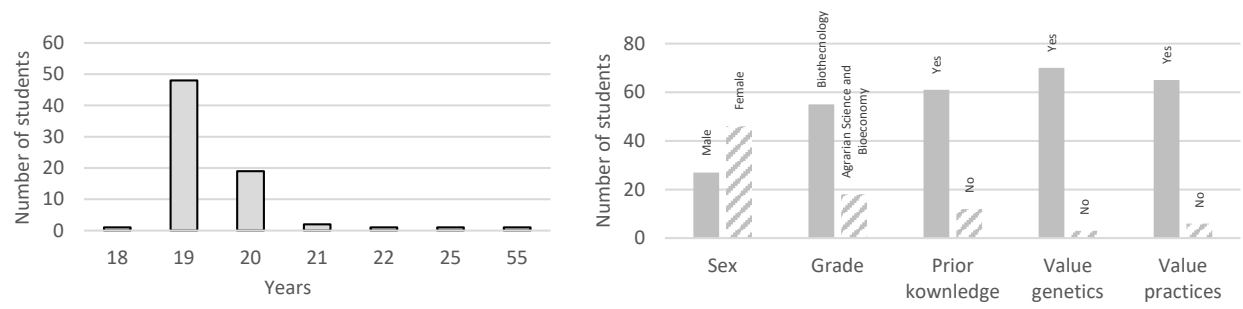

Figure S1. Students' profile. (a) Age range of students. (b) Personal and academic information.

Supplement: Supplementary file 1 [file plants-10-00694-s001.zip › Figure S1.pdf]
